# Vestibular Loss in Older Adults Is Associated with Impaired Spatial Navigation: Data from the Triangle Completion Task

**DOI:** 10.3389/fneur.2017.00173

**Published:** 2017-04-27

**Authors:** Yanjun Xie, Robin T. Bigelow, Scott F. Frankenthaler, Stephanie A. Studenski, Scott D. Moffat, Yuri Agrawal

**Affiliations:** ^1^Department of Otolaryngology-Head and Neck Surgery, Johns Hopkins University School of Medicine, Baltimore, MD, USA; ^2^Longitudinal Studies Section, Translational Gerontology Branch, National Institute on Aging, National Institute of Health, Baltimore, MD, USA; ^3^Department of Cognition and Brain Science, Cognitive Aging, School of Psychology, Georgia Institute of Technology, Atlanta, GA, USA

**Keywords:** triangle completion task, path integration, spatial navigation, visuospatial cognition, vestibular system, aging

## Abstract

**Background:**

Vestibular inputs have been shown to play a critical role in spatial navigation. In this study, we sought to evaluate whether vestibular loss due to aging contributes to impaired spatial navigation as measured by the triangle completion task (TCT).

**Materials and methods:**

We recruited three types of participants: young controls <55 years of age, older controls ≥55 years of age, and older patients from a Neurotology Clinic with evidence of vestibular physiologic impairment but who did not have any known vestibular disorder. We performed the cervical vestibular-evoked myogenic potential to evaluate saccular function and video head impulse testing to quantify horizontal semicircular canal vestibulo-ocular reflex gain. To assess spatial navigation ability, we administered the TCT, in which participants were conveyed along two segments of a pre-drawn triangular path and instructed to complete the final segment independently. We measured the angle (degrees) and distance (centimeters) of deviation from the correct trajectory. We evaluated the influence of vestibular inputs on TCT performance.

**Results:**

Forty-eight adults participated in the study (mean age: 62.0 years; 52.1% females), including 9 young controls, 15 older controls, and 24 clinic patients. Clinic patients had the greatest distance of deviation (67.7 cm), followed by older controls (45.4 cm), then young controls (27.8 cm; *p* < 0.01). Similarly, clinic patients had greater rotational angles (22.1°) compared to older (13.3°) and younger controls (12.4°; *p* < 0.01). Following multivariate linear regression adjusting for demographic variables, loss of otolith function was associated with an 18.2 cm increase in distance of deviation (95% CI: 15.2–47.4) and a 9.2° increase in rotational angle (95% CI: 3.0–15.5). Abnormal semicircular canal function was associated with a 26.0 cm increase in distance of deviation (95% CI: 0.2–51.8) and a 10.8° increase in rotational angle (95% CI: 3.0–15.5). Participants with both otolith and canal abnormalities had a larger distance error (β = 25.3, 95% CI: 6.2–44.4) and angle of deviation (β = 18.1, 95% CI: 10.1–26.2) than with either condition alone.

**Conclusion:**

Vestibular loss in older adults was associated with poorer performance on a dynamic spatial navigation task relative to old and young controls.

## Introduction

Spatial navigation is a fundamental cognitive motor activity that humans rely on for survival. Navigation strategies can be allocentric, i.e., reliant on external visual landmarks and environmental cues, or egocentric, i.e., using a person-centered frame of reference. Navigation also involves path integration, i.e., the reliance on self-motion cues provided by the visual system through optic flow, the proprioceptive system through feedback from muscles, tendons, and joints, and the vestibular system through the semicircular canal and otolith organs which sense angular and linear head acceleration, respectively (1, 2). Decline in sensory function has been associated with deficits in egocentric navigation (3). Specifically, individuals with vestibular loss have been shown to have difficulty with spatial navigation tasks such as walking a memorized route without visual feedback (4, 5).

Vestibular function declines with normative aging (6, 7). Older individuals with reduced vestibular function have been shown to have poorer performance on neurocognitive tests of spatial cognition, such as mental rotation tests or visual image retention tests (8). However, whether vestibular loss in aging adults is associated with impaired performance on dynamic spatial navigation tasks that provide real-time vestibular inputs has to our knowledge not been explored.

In this study, we evaluated performance on the triangle completion task (TCT) in a cohort of older adults with vestibular loss. The TCT, in which blindfolded participants are conveyed along two segments of a pre-drawn triangular path and instructed to complete the final segment independently, has been widely used as a test of path integration (9, 10). We recruited older patients presenting to a Neurotology Clinic with evidence of vestibular physiologic impairment but who did not have any known vestibular disorder such as Menière’s disease or acoustic neuroma. These individuals may have been particularly affected by vestibular loss risk factors that accumulate with age, such as ototoxic exposures, infections, and genetic predispositions (11). We compared the patients to two groups of controls: a healthy older control group and a young control group. We hypothesized that relative to young and older control participants, older individuals with reduced vestibular function will have greater impairment in path integration.

## Materials and Methods

### Study Participants

Study participants were aged 55 years and older and presented to the Johns Hopkins Neuro-Otology Clinic for dizziness. They had objective evidence of unilateral or bilateral vestibular impairment on vestibular physiologic testing as detailed below, and these participants had a negative clinical work-up for other vestibular pathologies including benign paroxysmal positional vertigo, Menière’s disease, acute or prior episodes of vestibular neuritis, superior canal dehiscence syndrome, acoustic neuroma, or an underlying neurologic disease that could cause dizziness. Thus, the Neurotology Clinic patients were considered to have aging-related decline in vestibular function (11, 12). Participants with unilateral vestibular loss also had a negative medical work-up (i.e., confirming no prior history of vestibular neuritis, superior canal dehiscence syndrome, or unilateral Menière’s disease, etc.) and were considered to have a unilateral form of aging-related vestibular loss. Among these participants, the etiology of their vestibular aging may be attributed to exogenous exposure(for example, ototoxic medication, previous infections), changes in the otoconial and macular structures of the inner ear, or intrinsic genetic susceptibilities that accumulate with age (11, 13).

Two types of control participants were recruited. Young controls were recruited from clinic staff <55 years. Older controls were ≥55 years and were recruited from the Baltimore Longi-tudinal Study of Aging (BLSA). The BLSA is an ongoing prospective cohort study of the normative aging process supported by the Intramural Research Program (IRP) of the National Institute on Aging (NIA). The study assesses the health, cognitive, and functional status of a cohort of over 1,300 relatively healthy community-dwelling individuals every 1–4 years. The BLSA takes place at the NIA IRP Clinical Research Unit in Baltimore, MD, USA.

Potential subjects in all settings were excluded if they could not participate in testing procedures due to blindness, poor neck range of motion or cervical spine instability, inability to walk unassisted, or memory or cognitive impairment. Individuals with conductive hearing loss were also excluded from air-conducted cervical vestibular-evoked myogenic potential (cVEMP) testing. All participants provided written informed consent. The study was approved by the Johns Hopkins Hospital Institutional Review Board, and the BLSA protocol was approved by the Institutional Review Board of the National Institute of Environmental Health Sciences in Research Triangle Park, NC, USA.

### Vestibular Testing

All participants underwent air-conducted cVEMP testing to assess saccular function (14) and video head impulse testing (vHIT) to assess horizontal semicircular canal function (15, 16). Details of both vestibular physiologic tests have been published previously. In brief, during cVEMP testing, participants sat upright and were instructed to flex their neck to provide tonic background muscle activity. Air-conducted 500 Hz (125 dB SPL) tone bursts were delivered monoaurally *via* intra-auricular speakers. Responses were recorded from an electrode montage consisting of a non-inverting electrode placed at the midpoint of the ipsilateral sternocleidomastoid muscle belly, an inverting electrode placed at the sternoclavicular junction, and a ground electrode placed on the manubrium sterni. Responses to 100 stimuli were averaged. The first positive and negative peaks that occurred between 13 and 23 ms after stimulus onset were designated p13 and n23. The raw peak-to-peak amplitude was calculated as the sum of the p13 and n23 amplitudes (in microvolts). The corrected peak-to-peak amplitude was calculated by dividing the raw amplitude by the rectified background EMG activity recorded during a 10-ms interval before the stimulus onset. The amplitude of the better-hearing ear was recorded for those with an intact cVEMP response. Individuals with no response above 100 dB acoustic clicks were considered to have absent function in the tested ear. In addition to recording continuous cVEMP amplitude measures, participants were categorized as having present, unilaterally absent, or bilaterally absent cVEMP response.

During the vHIT, participants removed their corrective spec-tacles and sat 1.25 m from a visual fixation target. They wore the EyeSeeCam video-oculography system consisting of a light-weight goggle frame with a built-in camera to record eye movements and an accelerometer to record head movements (Interacoustics USA, Eden Prairie, MN, USA). Right eye position was calibrated using projected targets from a glasses-mounted laser. A trained examiner tilted the participant’s head 30° below the horizon to bring the horizontal semicircular canal into the rotational plane. The examiner then applied 10–15 low amplitude (15°–20°) horizontal head impulses to each side with unpredictable direction and timing. Certified examiners evaluated vHIT tracings using custom software (MATLAB, MathWorks, Natick, MA, USA) and rejected tests with pupil tracking artifact or incorrect performance (i.e., low peak velocity or excessive head recoil or overshoot) (17). vHIT tracings were reviewed by trained examiners following well-established procedures in the senior author’s (YA) laboratory (7). Discrepancies and errors were adjudicated by the senior author. Peak head velocity ranged from 150°/s to 200°/s, and eye velocity was measured in the right eye from a two-point differentiator. Vestibulo-ocular reflex (VOR) gain was defined as the ratio of the area under the eye velocity curve to the head velocity curve from HIT onset until the head velocity returned to 0 (18). An abnormal VOR gain was defined as values <0.8 or >1.0. Participants were categorized as having normal, unilaterally abnormal, or bilaterally abnormal VOR gain. One BLSA participant had missing vHIT data, and four clinic patients had missing vHIT data. Vestibular physiologic impairment is defined by aberrant response in cVEMP testing or abnormal VOR gain in vHIT testing.

### Triangle Completion Task

The TCT was designed based on published procedures by Adamo et al., whose study team had previously developed this path integration task (19). Participants walked four triangular paths on a 10 ft × 10 ft flat tile floor in the testing area with their shoes on. Each triangle (92.5 cm × 185.5 cm × 212 cm) contained a 30°–60°–90° configuration. Before each trial, participants were instructed to look at the triangle they were about to walk on to become familiarized with the path. They were encouraged to complete a trial run using one triangular path with their eyes open. Then, participants were blindfolded and a noise-reducing headphone was placed on both ears to remove auditory input. As such, participants only had access to vestibular, proprioceptive and motor efference cues to perform the navigation task. The examiner stood side-by-side with the participant and supported his or her torso by placing hands lightly on the participant’s shoulders. In this fashion, participants were guided through two segments of the triangle and at the end of the second segment, they were asked to rotate on their own and walk along the hypotenuse back to the origin. Participants walked counterclockwise for the first two triangles and clockwise for the remaining two. For the first and third trials, participants started with the longer triangular limb (185.5 cm), and for the remaining trials, they started with the shorter triangular limb (92.5 cm). The midpoint that bisected the distance between two anterior tips of the feet was marked as the endpoint for each trial. The distance (in centimeters) that the participant deviated from the starting point (termed “*distance of deviation*”) and the absolute value of an angle (in degrees) the participant made with respect to the correct triangular limb (termed “*angle of deviation*”) were recorded with tape and retractable rulers.

### Data Analysis

Demographic, vestibular function, and performance on the TCT were analyzed using descriptive statistics. We performed a parametric analysis of variance (ANOVA) to compare each characteristic between young controls, older controls from the BLSA, and Neuro-Otology Clinic patients with physiologic evidence of vestibular function loss. In comparing VOR gain across participant groups, VOR gain of the “better ear” was used. Next, we used multivariate linear regression modeling pooling all participants to evaluate the association between vestibular parameters and TCT performance, after adjusting for demographic characteristics (age, sex, and race). A *p*-value of less than 0.05 was considered statistically significant. STATA 13 statistical software was used for all analyses (StataCorp, College Station, TX, USA).

## Results

Forty-eight individuals participated in the study (52.1% females), including 9 young controls (mean age: 30.8 years, SD: 6.3), 15 older controls (mean age: 69.1 years, SD: 12.2), and 24 older patients with dizziness (mean age: 69.3 years, SD: 10.1). Consecutive clinic patients fulfilling these criteria (~7% of patients presenting with dizziness) were recruited between January 2015 and March 2016 at the Johns Hopkins Hospital. Demographic, vestibular testing, and TCT performance data are presented in Table 1. None of the young controls had evidence of vestibular physiologic impairment. Two (13%) of 15 BLSA controls had abnormal cVEMP testing (both had bilaterally absent cVEMP responses), and 8 (57%) of BLSA controls had VOR gain abnormalities (5 unilaterally abnormal, 3 bilaterally abnormal). Among clinic patients, all patients had abnormal cVEMP testing: 50% had unilaterally absent cVEMP response and the remainder had bilaterally absent cVEMP responses. All but one clinic patient had abnormal VOR gain (13 unilaterally abnormal, 6 bilaterally abnormal). vHIT data were not available in one BLSA controls and four clinic patients due to participant refusal or exclusion criteria detailed in Section “Materials and Methods.” cVEMP amplitude was highest in young controls at 3.4 µV (SD: 0.8 µV) followed by 1.4 µV (SD: 0.6 µV) in older controls from the BLSA and 0.6 µV (SD: 0.3 µV) in clinic patients (*p* < 0.01 for overall ANOVA *F*-test). The VOR gain of the “better ear” did not significantly differ between the three groups of participants (*p* = 0.56). Similarly, average VOR gain between the left and right ears also did not significantly differ between the three groups of participants (*p* = 0.08). Audiometric data were available in 21 of 24 Neuro-Otology Clinic patients. Most clinic patients presented with mild to moderate high-frequency sensorineural hearing loss consistent with presbycusis. No participant presented with conductive hearing loss, and one presented with low-frequency sensorineural loss (subsequent work-up for Meniere’s disease was negative). The pure-tone average for these participants was 27.8 dB in the left ear and 32 dB in the right ear. In clinic patients, 8 of 11 patients with unilateral cVEMP abnormalities had bilateral high-frequency sensorineural hearing loss consistent with presbycusis (for the reminder, 1 participant had bilateral normal hearing and 2 had no audiometric data). Similarly, 7 of 13 patients with unilateral VOR gain abnormalities had bilateral high-frequency sensorineural hearing loss (for the remainder, 2 had bilateral normal hearing; 1 had unilateral low-frequency sensorineural loss in the contralateral ear and a negative work-up for Meniere’s disease; 3 individuals had no available audiometric data).

**Table 1:** **Demographic, vestibular function, and triangle completion task results**.^a^

	Young controls, *N* = 9	BLSA controls, *N* = 15	Neurotology Clinic patients, *N* = 24	*p* Value
Age (mean, SD)	30.8 (6.3)	69.1 (12.2)	69.3 (10.1)	**<0.01**
Sex (*n*, %)				
Male	3 (33.3)	9 (60.0)	11 (45.8)	
Female	6 (66.7)	6 (40.0)	13 (54.2)	0.45
Race (*n*, %)				
White	2 (22.2)	13 (86.7)	15 (62.5)	
African-American	3 (33.3)	1 (6.7)	5 (20.8)	
Other	4 (44.4)	1 (6.7)	4 (16.7)	**0.01**
Cervical VEMP (cVEMP) function category (*n*, %)				
Present	9 (100)	13 (86.7)	0	
Unilaterally absent	0	0	12 (50.0)	
Bilaterally absent	0	2 (13.3)	12 (50.0)	**<0.01**
cVEMP amplitude of the better ear (μV, SD)	3.4 (0.8)	1.4 (0.6)	0.6 (0.3)	**<0.01**
VOR gain category (*n*, %)				
Normal	8 (88.9)	6 (40.0)	1 (4.2)	
Unilaterally abnormal	1 (11.1)	5 (33.3)	13 (54.2)	
Bilaterally abnormal	0	3 (20.0)	6 (25.0)	0.15
VOR gain of the better ear (SD)	1.0 (0.0)	1.0 (0.1)	0.9 (0.3)	0.56
Distance of deviation (cm, SD)	27.8 (13.4)	45.4 (28.2)	67.7 (38.6)	**<0.01**
Angle of deviation (°, SD)	12.4 (4.5)	13.3 (12.9)	22.1 (13.1)	**0.04**

*^a^Young controls were <55 years old. Older controls were ≥55 years old from the Baltimore Longitudinal Study of Aging (BLSA). Dizzy patients were recruited from the Neurotology Clinic. Statistically significant results are bolded. For participants with unilaterally absent cVEMP response, cVEMP parameters from the remaining ear were included. Individuals with bilaterally absent cVEMP responses were excluded from the cVEMP amplitude calculations*.

*cVEMP, cervical vestibular-evoked myogenic potential; VOR, vestibulo-ocular reflex*.

In the TCT, we observed a stepwise increase in the distance of deviation between young controls (27.8 cm), older controls (45.4 cm), and older, dizzy patients (67.7 cm; *p* < 0.01 for ANOVA *F*-test). For the rotational angle, both young (12.4°) and older controls (13.3°) deviated less than clinic patients (22.1°; *p* < 0.01 for ANOVA *F*-test).

**Figure 1:**
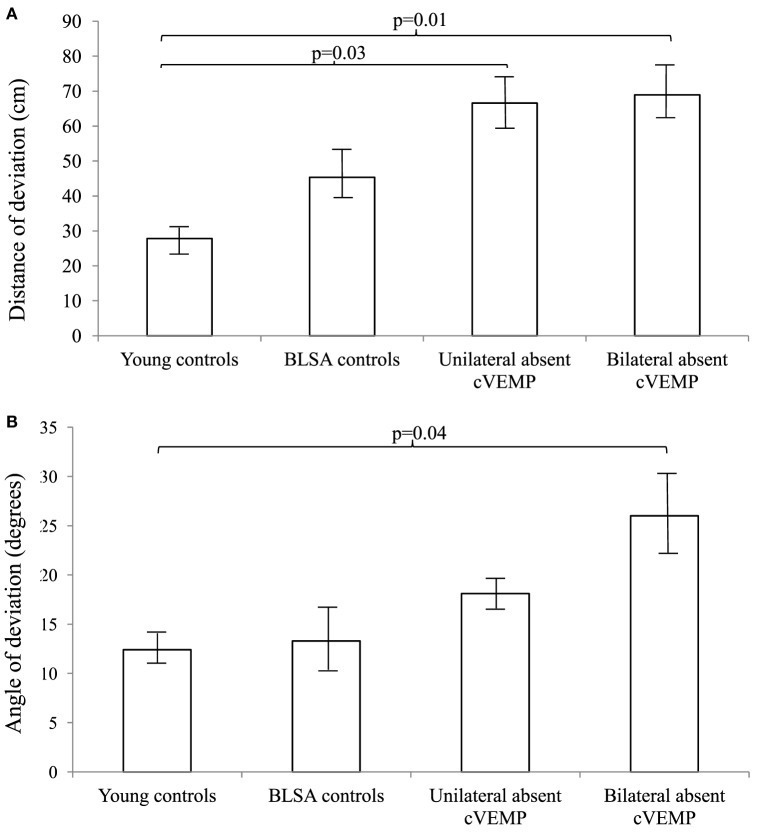
**Distance (A) and angles (B) of deviation by cervical vestibular-evoked myogenic potential (cVEMP) response**. We included 9 young controls, 15 Baltimore Longitudinal Study of Aging (BLSA) controls, and 24 clinic patients (12 unilateral absent and 12 bilaterally absent cVEMP responses). An analysis of variance demonstrated significant differences in the distance and angles across cVEMP categories (*p* < 0.01). A pairwise *post hoc* analysis revealed differences that were indicated by the brackets. Error bars indicate the SEM.

**Figure 2:**
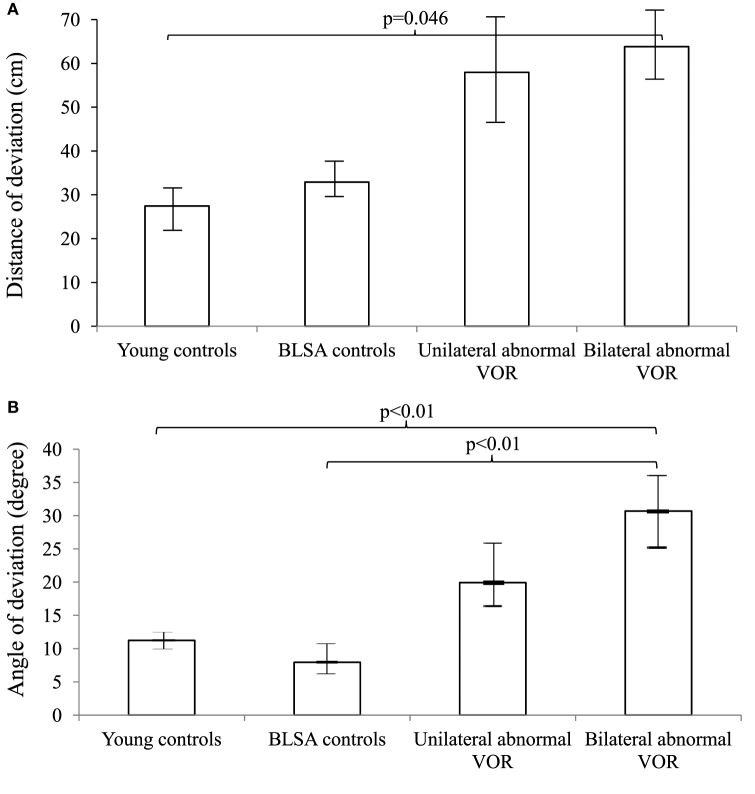
**Distance (A) and angles (B) of deviation by vestibulo-ocular reflex (VOR) gain**. We included 9 young controls, 15 Baltimore Longitudinal Study of Aging (BLSA) controls, and 19 Neuro-Otology Clinic patients (13 unilateral abnormal and 6 bilaterally abnormal VOR gain). An analysis of variance (ANOVA) demonstrated differences in the distance and angles across VOR categories (*p* = 0.03 for the distance and *p* < 0.01 for the angle). A pairwise *post hoc* analysis revealed differences that were indicated by the brackets. Error bars indicate the SEM.

We further categorized all participants into groups according to whether they had normal, unilaterally abnormal, or bilaterally abnormal cVEMP and VOR gain responses (Figures 1 and 2). As such, control participants with vestibular impairment were categorized with clinic patients in the abnormal vestibular response groups. For cVEMP responses, we observed that as cVEMP function worsened, the distance and angle of deviation increased. For VOR gain, we observed a similar relationship such that as VOR gain function worsened, both the distance and angle of deviation became larger. *Post hoc* pairwise comparison tests were performed to further analyze these differences, and statistically significant results are represented in Figures 1 and 2.

Next, we used multivariate linear regression models to evaluate the association between vestibular function (cVEMP and VOR gain) and the distance and angle of deviation across all participants (Table 2). To simplify these analyses, we collapsed the unilaterally and bilaterally abnormal categories into a single abnormal category for both cVEMP and VOR gain responses. We also created a category of participants who had concurrent cVEMP and VOR abnormalities. In analyses adjusted for age, sex and race/ethnicity, we observed that an abnormal cVEMP was associated with an 18.2 cm increase in deviation distance [95% confidence interval (95% CI): 15.2–47.4] and a 9.2° increase in the rotational angle (95% CI: 3.0–15.5). The cVEMP amplitude was not associated with the distance or angles of deviation, although it should be noted that participants with absent cVEMP responses (*N* = 14) were not included in this analysis. Similarly, an abnormal VOR response was associated with a 26.0 cm increase in the distance of deviation (95% CI: 0.2, 51.8) and a 10.8° increase in the angle of deviation (95% CI: 3.0, 15.5). The VOR gain value was also not associated with the distance or angle of deviation. Of note, age was a significant independent predictor of distance but not angle of deviation (data not shown). Finally, participants with both cVEMP and VOR abnormalities (*N* = 13) on testing had a significantly larger distance of deviation (β = 25.3, 95% CI: 6.2–44.4) and rotational angle (β = 18.1, 95% CI: 10.1–26.2) than with either vestibular abnormality alone in adjusted analyses.

**Table 2:** **Multivariate linear regression models of the association between vestibular function and triangle completion task performance in all participants**.^a^

	Distance of deviation (cm)		Angle of deviation (°)	
	Unadjusted β (95% CI)	Adjusted β (95% CI)	Unadjusted β (95% CI)	Adjusted β (95% CI)
**cVEMP response**				
Abnormal cVEMP response	**31.3 (15.2, 47.4)**	**18.2 (1.2, 35.1)**	**11.7 (6.1, 17.3)**	**9.2 (3.0, 15.5)**
cVEMP amplitude of the better ear (μV)	−5.4 (−13.9, 3.1)	−9.6 (−20.4, 1.2)	−0.5 (−3.1, 2.1)	−2.1 (−5.3, 1.1)
**VOR gain**				
Abnormal VOR gain	27.4 (−0.5, 55.1)	**26.0 (0.2, 51.8)**	**11.2 (1.2, 21.2)**	**10.8 (0.9, 20.7)**
VOR gain of the better-hearing ear	−14.7 (−69.6, 40.1)	−38.3 (−92.6, 16.0)	−11.0 (−36.8, 14.8)	−15.4 (−42.8, 12.1)
**cVEMP and VOR function**				
Concurrent cVEMP and VOR abnormality	**33.5 (17.4, 49.7)**	**25.3 (6.2, 44.4)**	**16.3 (9.4, 23.1)**	**18.1 (10.1, 26.2)**

*^a^Models were adjusted for demographic covariates (age, sex, race/ethnicity). An abnormal cVEMP response includes unilaterally or bilaterally absent cVEMP. Likewise, an abnormal VOR gain includes unilaterally or bilaterally abnormal VOR gain value (<0.8 or >1). Statistically significant regression models are bolded*.

*cVEMP, cervical vestibular-evoked myogenic potential; VOR, vestibulo-ocular reflex; 95% CI, 95% confidence interval*.

## Discussion

In this study, we found that older individuals with vestibular physiologic loss had impaired egocentric spatial navigation, as measured by the TCT. We evaluated three groups of participants: older clinic patients with vestibular loss, older controls from the BLSA, and young controls. We observed that both groups of older participants deviated more and made larger rotational angle errors compared to young controls. In addition, older clinic patients had greater impairment in the TCT relative to the older control cohort. Consistent with this finding, we found that absent cVEMP responses and abnormal VOR gain were significantly associated with larger distance error and greater angle of deviation. Moreover, the greatest impairment in TCT performance occurred in individuals with both abnormal cVEMP and VOR gain. Altogether, these findings support several inferences. First, the data corroborate the importance of vestibular information for path integration. Second, vestibular deficits that accrue as a result of aging contribute to navigational impairment, as evidenced by the substantial impairment present in the older clinic patients with vestibular physiologic loss. Third, other factors beyond clinical vestibular loss also appear to contribute to impaired egocentric navigation, as demonstrated by the healthy older controls with relative preservation of vestibular physiologic function but with greater navigational impairments compared to young controls. These factors may include subclinical vestibular loss, proprioceptive impairment, and decline in the sensitivity to motor efference information.

Our study confirms and extends prior reports of impaired egocentric spatial navigation in labyrinthine-defective patients. A previous study by Glasauer et al. using a similar triangle completion paradigm in 5 patients with vestibular loss due to acoustic neuroma resection or bilateral vestibular ototoxicity found that the patients made significantly greater rotation angle errors though not distance errors compared to both young and age-matched controls (5). Interestingly, the investigators had previously observed that patients with bilateral labyrinthine deficiency did not make navigation errors on a task that involved walking toward a memorized target along a simple linear trajectory (20). Glasauer and colleagues proposed that vestibular information is particularly critical to navigating across more complex trajectories involving rotations and the need for angular velocity estimation. In another study, Adamo et al. investigated age-related differences in TCT performance among healthy individuals and reported that older compared to younger individuals showed greater errors in angle rotation estimations (19). Our study extends these two prior reports in finding that both vestibular loss and age are independent contributors to spatial navigation ability.

The hippocampus and entorhinal cortex are thought to be sites where a cognitive map of space is maintained by special populations of place cells and grid cells, respectively (21). Studies suggest that the vestibular system provides critical inputs to the generation of the cognitive map. In animal models, peripheral vestibular stimulation has been shown to result in increased firing of hippocampal cholinergic neurons (22). Selective lesion of the peripheral vestibular system in rats has also been shown to lead to a loss of specificity of hippocampal place cell firing (23). More-over, so-called “head direction cells” present in the subiculum, thalamus, and other regions fire when an animal faces a particular direction, and are known to be dependent on vestibular input (24). Most importantly for the present purposes is the observation that the head direction system codes for heading accuracy (and error) in a non-visual path integration task (25). In humans, a study of 10 patients who underwent bilateral vestibular deafferentation found that the patients exhibited impaired spatial navigation skills and reduced hippocampal volumes relative to age-matched controls (26). In the current study, we sought to further evaluate whether age-related vestibular loss influences performance of a dynamic spatial navigation task. This task may be a better proxy for real-world navigational tasks, such as driving, wayfinding, and route-learning which are known to deteriorate with age (27). Our data suggest that vestibular loss associated with aging may contribute to these navigational impairments in older adults. Combined with the results from studies in non-human species, the loss or reduction in vestibular input could affect both the place and head direction systems and impair path integration and navigation performance in older adults.

In a recent work on a cohort of healthy older adults, we observed that poorer vestibular function was associated with reduced spatial cognitive skills (8, 27, 28). These studies involved pencil and paper-based neurocognitive tests of spatial cognitive skills, such as mental rotation and visual image retention. These tests were administered with the subject seated and did not involve real-time vestibular stimulation. We observed that both semicircular canal and otolith deficits are associated with spatial navigation impairment and that individuals with both semicircular canal and otolith deficits had the poorest performance on the TCT. These findings suggest that both vestibular end-organs contribute independently to spatial navigation ability. The semicircular canals detect angular rotations of the head, and not surprisingly, semicircular canal function was significantly associated with the angle and distance of deviation. The otolith organs sense linear head translations and the orientation of the head with respect to gravity. In prior work in healthy older adults, we observed that otolith (specifically saccular) function was significantly associated with spatial memory and mental orientation performance (8). While the canals may be important for sensing and computing angular movements, the otoliths may provide a constant input to the brain about head orientation which is used to generate a cognitive map of the external environment.

We note that several participants in the BLSA control group had cVEMP and/or VOR gain abnormalities. These older subjects from the Baltimore Longitudinal Study of Aging are healthy individuals with no complaints of vertigo or imbalance. It is interesting that 2 (13.3%) BLSA controls had abnormal cVEMP, while 8 (53.3%) individuals had VOR gain abnormality. In a previous study, we observed that more than 50% of BLSA participants >60 who underwent vHIT had compensatory saccades, which were indicative of poor VOR function (29). Moreover, we observed in the same study that the prevalence of compensatory saccades was linked to VOR gain. In the present study involving more than 50% of BLSA controls with abnormal VOR gain, these findings may represent a subclinical decline of vestibular function in older adults. Future studies are needed to understand the significance of abnormal vestibular findings in older adults with no subjective vertigo or balance complaints.

We note the following limitations of our study. The study was cross-sectional; therefore, causal inferences about the relationship between vestibular function and TCT performance cannot be made. Additionally, our older clinic patients with vestibular physiologic loss were assumed to have this loss attributable to age, given that they did not have any other specific vestibular disorders. However, given that age-related vestibular loss is a diagnosis of exclusion, this cannot be definitively confirmed. We have currently added the TCT to the BLSA test battery and will be able to test a substantially larger sample of older adults with greater variation in vestibular function from which we will be able to more clearly evaluate the relationship between age-related vestibular loss and spatial navigation. Secondly, horizontal VOR gain and cVEMP response were used to assess horizontal semicircular canal and saccular function, respectively. Functions of the remaining anterior and posterior semicircular canals (evaluated by vertical vHIT) and utricle (evaluated by ocular VEMP testing) would be informative. These tests were not performed given the time and participant burden constraints with healthy subjects in the Baltimore Longitudinal Study of Aging (30).

Furthermore, we observed several cases of VOR gain >1.0 in both BLSA controls and clinic patients. In our previous study of BLSA participants, we also observed similar rates of super-unity gains among the BLSA participants and found that super-unity gains were significantly associated with the presence of “back-up” compensatory saccades (i.e., in the anti-compensatory direction). These data suggest that the super-unity gains may not simply be artifactual from goggle slippage. Nevertheless, we recognize several mechanisms by which super-unity VOR gains may be observed, including cerebellar disinhibition, use of magnifying spectacles for the correction of presbyopia, as well as artifact from goggle slippage (29). In our testing, we tried to apply the goggles as tightly as we thought possible around the participants’ head while maintaining their comfort. Nevertheless, slippage may have occurred. Finally, spatial navigation relies on multiple sensory inputs as well as judgment of motor output. We attempted to independently assess vestibular input to navigation by eliminating visual and hearing cues during the TCT. In the present study, we did not include patients with inability to walk unassisted. A recent study showed that beyond the common influence of age, sensorimotor declines appeared to occur independently (31). As such, we expected that including age in the regression models should account for much of the other potential deficits that may occur in participants. However, we acknowledged that we could not exclude proprioceptive or motor efference input during the TCT, and thus cannot definitively rule out their contribution to performance of the TCT. We selected the TCT protocol described because it is easily administered in the clinical setting and a reasonable first step for testing our hypothesis about the influence of age-related vestibular loss on spatial navigation. Further work is in progress in our group using other tests, including chair rotation tests, which evaluate spatial cognition in a condition involving active vestibular stimulation but while also eliminating proprioceptive and motor efference inputs.

In summary, we observed that vestibular loss associated with age contributed to impaired spatial navigation, as measured by angle and distance of deviation on the TCT. Both semicircular canal and otolith function were significantly associated with TCT performance, and abnormality in both semicircular canal and otolith function was associated with poorest performance. Vestibular loss in aging adults may contribute to the difficulties older adults experience with real-life navigation tasks, including driving, wayfinding, and route-learning, which substantially limit their autonomy and quality of life (32, 33).

## Ethics Statement

This study was carried out in accordance with the recommendations of the Johns Hopkins Hospital Institutional Review Board and the Institutional Review Board of the National Institute of Environmental Health Sciences in Research Triangle Park with written informed consent from all subjects. All subjects gave written informed consent in accordance with the Declaration of Helsinki. The protocol was approved by the Johns Hopkins Hospital Institutional Review Board and the Institutional Review Board of the National Institute of Environmental Health Sciences.

## Author Contributions

YX: substantial contribution to the conception and design of the work; acquisition, analysis, and interpretation of the data; drafting the manuscript and revising it critically for important intellectual content; final approval of the version to be published; agreement to be accountable for the work. RB and YA: substantial contribution to the conception and design of the work; acquisition, analysis, and interpretation of the data; revising the work critically for important intellectual content; final approval of the version to be published; agreement to be accountable for the work. SF: acquisition, analysis, and interpretation of the data; revising the work critically for important intellectual content; final approval of the version to be published; agreement to be accountable for the work. SS and SM: substantial contribution to the conception and design of the work; analysis and interpretation of the data; revising the work critically for important intellectual content; final approval of the version to be published; agreement to be accountable for the work.

## Conflict of Interest Statement

The authors declare that the research was conducted in the absence of any commercial or financial relationships that could be construed as a potential conflict of interest.
